# E-cigarettes and smoking cessation: a critique of a New England Journal Medicine-commissioned case study

**DOI:** 10.1007/s11739-016-1537-0

**Published:** 2016-09-24

**Authors:** Riccardo Polosa, Pasquale Caponnetto

**Affiliations:** 1 Centro Prevenzione e Cura del Tabagismo, Azienda Ospedaliero Universitaria “Policlinico-V. Emanuele”, Catania, Italy; 2 Dipartimento di Medicina Clinica e Sperimentale, Università di Catania, Catania, Italy; 3 UOC di Medicina Interna e d’Urgenza, Azienda Ospedaliero Universitaria “Policlinico-V. Emanuele”, Catania, Italy

**Keywords:** Smoking cessation, Smoking reduction, Electronic cigarette, Tobacco harm reduction

A recent article in the NEJM [1] illustrates the case vignette of a male smoker being seen at a routine health check-up that prompted him to enquiry about quitting using e-cigarettes.

He is 29 years old, but with a significant medical history of obesity (current BMI = 31), hypertension (current BP is 128/76 mmHg on chlorthalidone 25 mg daily) and childhood seizures. The rest of the physical examination and the review of his systems were unremarkable.

The patient started smoking when he was 15 years old, and he has been smoking up to 1.5 packs per day for the past 6 years. He has reduced or stopped smoking several times in the past using various nicotine replacement therapies (NRTs) or by quitting “cold turkey”. On each occasion, he was able to sustain his efforts for up to 3 weeks before relapsing into his previous smoking habits.

There follows a discussion about the danger of smoking and potentially useful smoking cessation aids. The patient has friends who have stopped smoking cigarettes by switching to regular use of e-cigarettes. He is now interested in giving up smoking by trying e-cigarettes, and asks for an opinion.

The Editorial staff at the NEJM invites two experts to provide their personal recommendations.

Expert no. 1 is Christopher Bullen, a professor of Public Health at the Faculty of Medical and Health Sciences of the University of Auckland. His research interests focus primarily on tobacco control and innovative smoking cessation interventions research, including e-cigarettes. He also has wider interests in research on population health education, and heart disease prevention and treatment. He directs the National Institute for Health Innovation (NIHI).

He recommends trying e-cigarettes for smoking cessation because he thinks that the patient has limited pharmacologic treatment options (contraindications for bupropion and varenicline prescription due to his history of seizures; inability to abstain from smoking on several occasions despite being on NRT).

Expert no. 2, is Stanton Glantz, a professor of Medicine in the Division of Cardiology, and American Legacy Foundation Distinguished Professor of Tobacco Control. He conducts research on a wide range of topics ranging from the health effects of second hand smoke (with particular emphasis on the cardiovascular system) to the efficacy of different tobacco control policies. His work has recently expanded to include e-cigarettes. He directs the Center for Tobacco Control Research and Education at the University of California, San Francisco (UCSF) School of Medicine.

He tries to convince the patient that he should reconsider his interest in giving up smoking by trying e-cigarettes by applying a motivational approach based on the results of a—flawed—meta-analysis showing that these products are not proven to assist smoking cessation [2]. The meta-analysis took studies that contained no useful information on whether vaping helps smokers to quit, and misreported them as if they were showing that vaping undermines quitting. These were primary studies that compared smokers who tried vaping, but continued to smoke with smokers who did not try vaping. Smokers who tried vaping and successfully stopped smoking were excluded, so treatment efficacy was evaluated only on treatment failures. In relation to the patient’s case, he does not provide any solution besides suggesting to stop using conventional cigarettes (easier said than done).

We (Dr. R. Polosa and Dr. P. Caponnetto) run one of Italy’s busiest smoking cessation centers, and have helped thousands of smokers who successfully quit with several methods including e-cigarettes. We were the first in the world to publish a smoking cessation study, a RCT, and the longest clinical follow-up with e-cigarettes. According to a recent bibliometric analysis [3], we have been ranked as the top most prolific authors who published in the field of e-cigarettes.

We were pleased to see featured this case study of a 29-year-old man interested in giving up smoking by switching to e-cigarettes in the NEJM, and we would like to offer our personal opinion, bringing into the debate the combined experience of an experienced internist (R. Polosa) with that of a clinical psychologist who is in charge of multimodal smoking cessation programs (P. Caponnetto).

The case study presented is more challenging than actually appears for several reasons.

The patient is relatively young, and quit rates in young adults are known to be very low as proven by his history of frequent relapses. There is no evidence demonstrating the efficacy of FDA-approved smoking cessation drugs for young adults. Thus, FDA-approved smoking cessation drugs cannot be recommended for young adults.

The patient’s history indicates that he is at high risk of relapse. While international guidelines place great emphasis on relapse prevention, very little can be done to manage smokers with a history of frequent relapses [4]. If the patient is willing, increasing the intensity and frequency of cessation counseling or adding another first line medication may increase success rates. Nevertheless, as mentioned earlier, there is no evidence to support the recommendation of smoking cessation medications for young adults who smoke.

The patient is already overweight, and is being treated for hypertension. Stopping smoking is known to lead to weight gain in four out of five quitters. Hence, it is important to consider that he—if successful—will have to deal with the burden of post-cessation weight gain with its important negative health consequences, particularly in consideration of the fact that obesity and hypertension are well known risk factors for cardiovascular disease.

While there is no doubt that stopping smoking will result in considerable health improvements, the patient requires an alternative approach. An additional option is to encourage him to switch to a much cleaner source of nicotine. Given that his personal preference is to try e-cigarettes to quit smoking, his choice should be respected, and the health care provider should offer a balanced overview of their risk/benefit ratio [5].

More specifically, relevant to this patient’s clinical history, it is worth noticing that (1) e-cigarettes have helped abstaining from conventional cigarette young adults as well as older ones [6]; (2) e-cigarettes have been shown to reduce post-cessation weight gain in quitters [7]; (3) smokers with elevated blood pressure who quit by switching to e-cigarettes may lower their BP in the long-term [8].

Physicians should advise smokers about the most effective ways to quickly reduce their risk. While smoking cessation may be the most desirable final outcome from a health point of view, it may be the wrong goal if it leads to failure or relapse. Physicians should consider all the pathways available to a smoking patient, and select the ones that give the greatest probability of eliminating exposure to tobacco smoking, including e-cigarettes. There is now increasing evidence that—for many smokers—the best outcome may be a long-term switch to vaping, tolerating the small residual risk in return for a higher likelihood of success.
